# Iodide of Potassium in Asthma

**Published:** 1886-07

**Authors:** 


**Iodide of Potassium in Spasmodic Asthma.**—Dr. J. Ormford publishes in the *Practitioner* (April 10, 1886) a tabular analysis of the results obtained in thirty-six cases of asthma treated with iodide of potassium. All of the cases displayed, though with varying severity, the cardinal symptoms of the disease,—that is, difficulty of breathing coming on suddenly, usually in the early morning during sleep, and passing off after a time, so as to leave the patient comparatively well, but recurring usually in a regular fashion at regular intervals. The iodide was given alone, or, if in combination, only after the effect of the uncombined drug had been tried, and it proved a failure only in nine cases out of thirty-six,—that is, in twenty-five per cent., while its good effects were not limited to the uncomplicated cases. Five or ten grains three times a day, seemed to suit best in most cases, but in some a larger or smaller dose did better. In some, again, an increase of the dose did good for a time, but the effects seemed to wear off. In confirmed cases the drug can hardly be claimed to be curative, though it will in many relieve attacks, which, however, are apt to return when the use of the drug is stopped.

